# Phase‐Changing Microcapsules Incorporated with Black Phosphorus for Efficient Solar Energy Storage

**DOI:** 10.1002/advs.202000602

**Published:** 2020-10-22

**Authors:** Hao Huang, Tongyu Shi, Rui He, Jiahong Wang, Paul K. Chu, Xue‐Feng Yu

**Affiliations:** ^1^ Shenzhen Engineering Center for the Fabrication of Two‐Dimensional Atomic Crystals Shenzhen Institutes of Advanced Technology Chinese Academy of Sciences Shenzhen 518055 P. R. China; ^2^ University of Chinese Academy of Sciences Beijing 100049 P. R. China; ^3^ Department of Physics Department of Materials Science and Engineering, and Department of Biomedical Engineering City University of Hong Kong Tat Chee Avenue, Kowloon Hong Kong China

**Keywords:** black phosphorus, microcapsules, phase‐changing materials, photothermal materials, solar energy

## Abstract

A new solar energy storage system is designed and synthesized based on phase‐changing microcapsules incorporated with black phosphorus sheets (BPs). BPs are 2D materials with broad light absorption and high photothermal performance, which are synthesized and covalently modified with poly(methyl methacrylate) (PMMA) to produce the PMMA‐modified BPs (mBPs). With the aid of PMMA, the mBPs and phase‐changing materials (PCM, eicosane) are encapsulated together to form microcapsules. The microencapsulated eicosane and mBPs (mBPs‐MPCM) composites exhibit a high latent heat of over 180 kJ kg^−1^, good thermal reliability, as well as excellent photothermal characteristics inherited from BPs. Owing to the direct contact in the integrated mBPs‐MPCM composites, the thermal energy generated by mBPs is transferred to eicosane immediately giving rise to three times higher efficiency in solar energy storage compared to microcapsules with mBPs on the surface. The mBPs‐MPCM composites have great potential in solar energy storage applications and the concept of integrating photothermal materials and PCMs as the core provides insights into the design of high‐efficiency solar energy storage materials.

## Graphical abstract

Phase‐changing microcapsules incorporated with black phosphorus are designed and prepared for efficient solar energy storage. Because of the direct contact between the black phosphorus sheets and eicosane, the microencapsulated composites show reduced energy loss during solar‐thermal energy transfer and accelerated solar energy storage. This structure has large potential in high‐efficiency solar energy storage.

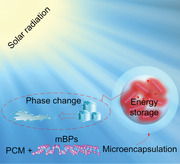

Solar energy is one of the most important renewable energy sources in the continuous efforts to meet the ever‐increasing global energy demand^[^ ^1^, ^2^ ^]^ and techniques that capture solar energy have attracted enormous attention, especially harvesting of solar energy for photovoltaic applications.^[^ ^3^, ^4^, ^5^, ^6^, ^7^ ^]^ In particular, solar‐thermal processes are especially appealing to seawater desalination, space heating, construction, and solar‐thermal storage systems.^[^ ^8^, ^9^, ^10^ ^]^ However, solar radiation varies from time to time and place to place. In this respect, phase‐changing materials (PCMs) with a large latent heat and heat storage density are considered efficient materials to resolve the time mismatch between the heat supply and actual consumption because PCMs can be exploited to store and release energy as a result of the phase change.^[^ ^11^ ^]^ Compared to inorganic PCMs, organic PCMs have many practical virtues such as the good chemical stability, thermal reliability, little super‐cooling, and reasonable cost.^[^ ^12^ ^]^ However, the low thermal conductivity and leakage problem have hitherto stifled wider application of organic PCMs.^[^ ^13^ ^]^

Microencapsulated PCMs (MPCMs) composites have aroused much interest because they possess a PCM core for energy storage and shell structure to prevent leakage of PCMs during the phase change.^[^ ^13^, ^14^ ^]^ Much effort has been made to improve MPCM composites by, for example, blending with photothermal materials and microencapsulating techniques.^[^ ^15^, ^16^, ^17^, ^18^, ^19^ ^]^ Generally, the photothermal materials function as the solar collector to absorb and convert solar energy into thermal energy and the generated energy is then transferred to the PCMs via thermal diffusion. Most of the research activities have focused on combining additional materials with the shell structure.^[^ ^15^, ^16^, ^17^ ^]^ However, a lot of thermal energy is wasted in the heat flow because the shell raises the heat resistance between the photothermal materials and PCMs core consequently impeding transfer of thermal energy to the inner PCMs.

Herein, a highly efficient solar energy storage system is designed with polymethyl methacrylate (PMMA), a high light‐transmittance polymer, as the compact shell and organic PCM (eicosane) together with PMMA‐modified black phosphorus sheets (mBPs) as the core. Black phosphorus (BP) is a promising 2D layered structure suitable for electronic and optoelectronic devices due to the unique band structure, high charge carrier mobility, and ambipolar conduction ability.^[^ ^20^, ^21^ ^]^ Particularly, the tunable bandgap of BPs in the range of 0.3–2.0 eV enables light absorption spanning the ultraviolet (UV) and near‐infrared (NIR) regions.^[^ ^22^ ^]^ It has been shown that layered BPs possess high photothermal conversion efficiency and large extinction coefficients^[^ ^23^ ^]^ thereby rending BPs promising as solar‐thermal materials. Actually, the BPs have been used in the photovoltaic device and solar cells for solar energy storage because of the unique photoelectronic properties.^[^ ^24^, ^25^ ^]^ However, to the best of our knowledge, the combination of BPs with PCMs for solar energy storage has not been reported yet. In this work, by taking advantage of the high photothermal conversion efficiency of BPs, the microencapsulated eicosane and mBPs (mBPs‐MPCM) composites show reduced energy loss during solar‐thermal energy transfer and accelerated solar energy storage. Our study of mBPs‐MPCM composites reveals a new and efficient approach to integrate photothermal materials and PCMs and expedite the application to solar energy storage.

The BPs are exfoliated and modified with PMMA by a one‐step process to improve the dispersibility in the oil‐phase (solvent CH_2_Cl_2_). Briefly, the mBPs are prepared with PMMA molecules by a tip sonication process (Figure S1, Supporting Information). The PMMA is dissolved in CH_2_Cl_2_ and mixed with BP powder in the *N‐*methyl‐2‐pyrrolidone (NMP) solution followed by tip sonication. The scanning electron microscopy (SEM) image and transmission electron microscopy (TEM) image in **Figure** **1**a,b show that the mBPs have a lateral size of several hundred nanometers. The inset in Figure 1b reveals a clear lattice spacing of ≈0.21 nm (belongs to (002) plane of orthorhombic phosphorus, ICDD–PDF 76–1963) and high crystalline quality. As shown in the atomic force microscopy (AFM) result in Figure 1c, the thickness of the mBPs varies from 6 to 10 nm which is comparable with bare BPs (Figure S2, Supporting Information). Fourier transform infrared (FTIR) spectroscopy and X‐ray photoelectron spectroscopy (XPS) are performed to confirm modification of PMMA on BPs. As shown in Figure 1d, mBPs and pure PMMA exhibit the two typical peaks of C=O at 1730 cm^−1^ and C—O group at 1243 cm^−1[^ ^26^, ^27^ ^]^ but in contrast, the bare BPs prepared without PMMA shows no characteristic peaks of PMMA. The high‐resolution XPS spectra of the bare BPs and mBPs and deconvoluted spectra are displayed in Figure 1e. The three C 1s peaks at 284.6, 286.3, and 288.8 eV of the two samples can be assigned to residual solvent molecules, whereas the large peaks at 286.0 eV and 288.6 eV observed from mBPs arise from C—O and C=O in the PMMA, respectively.^[^ ^28^ ^]^ The results corroborate successful modification of PMMA on BPs. As shown in Figure S3, Supporting Information, crystalline BP bands of P 2p1/2 (130.5 eV) and P 2p3/2 (129.5 eV) are observed from the samples but the sub‐bands of the oxidized phosphorus (P*_x_*O*_y_*) are different. In the XPS spectrum of mBPs, the sub‐bands can be assigned to P*_x_*O*_y_* (133.6 eV) and P—O—C (134.5 eV)^[^ ^29^, ^30^ ^]^ conveying that there are covalent bonds between the PMMA molecules and BPs. The O 1s spectra of bare BPs (Figure S4, Supporting Information) show bands of P=O (532.2 eV) and P—O—P (533.3 eV), indicating the high chemical activity of BP during ultrasonic process under non‐strict oxygen‐free condition which may promote the interaction between the PMMA molecules and BPs. The mBPs are dispersed well in CH_2_Cl_2_ and show a broadband absorption range spanning the UV and NIR regions as the bare BPs in NMP ((Figure 1f and Figure S5, Supporting Information), but on the other hand, the bare BPs aggregate promptly in CH_2_Cl_2_ (Figure 1f). Also, the good dispersibility of mBPs in oil‐phase containing PMMA, eicosane, and CH_2_Cl_2_ can be observed (Figure S6, Supporting Information).

**Figure 1:**
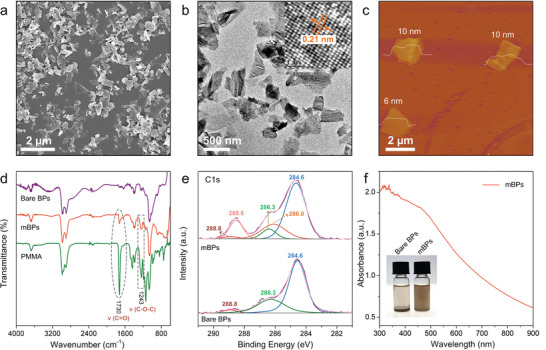
a,b) SEM and TEM images (HR‐TEM image shown in the inset) of the mBPs; c) AFM image of the mBPs; d) FTIR spectra of the bare BPs, mBPs, and PMMA; e) XPS C 1s spectra of the bare BPs and mBPs; f) Absorption spectrum of the mBPs with the inset showing the photographs of the bare BPs and mBPs in CH_2_Cl_2_.

The synthetic process of the mBPs‐MPCM composites is illustrated in **Figure** **2**. Polyvinyl alcohol (PVA) is dissolved in hot water to form a water‐phase solution and owing to the improved dispersibility of mBPs, the oil‐phase solution is prepared by mixing the mBPs, PMMA, and eicosane in CH_2_Cl_2_. The oil‐phase solution is then added to the water‐phase solution under vigorous stirring to form a stable emulsion. After evaporation of CH_2_Cl_2_, the PMMA approaches the oil–water interface and polymerizes to form a shell structure, while the mBPs are retained with eicosane as the core materials.

**Figure 2:**
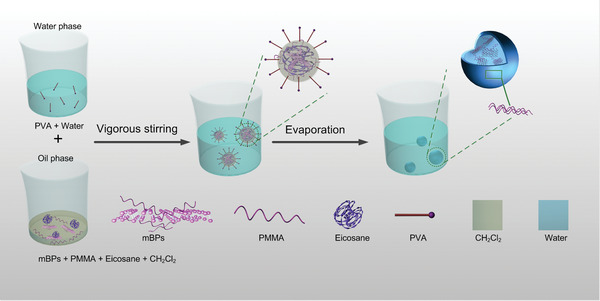
Schematic illustration of the synthesis of the mBPs‐MPCM.

A series of experiments involving different core/shell ratios are performed to optimize the encapsulation procedures. As shown in **Figure** **3** and Figure S7, Supporting Information, SEM is employed to monitor the morphological evolution of the microcapsules. The MPCM composites exhibit a spherical shape and clean surface (Figure 3a and Figures S7a,b, Supporting Information). If the mass ratio of the core/shell is increased to 13.3, the microcapsules collapse (Figure S7c, Supporting Information) indicating lower encapsulation efficiency of the core. On the other hand, the MPCM composites retain the shape when the mass ratio of the core/shell is reduced to 4.4 (Figures S7d–f, Supporting Information). Accordingly, an optimal mass ratio of 8 is adopted to prepare the MPCM composites. The mBPs‐MPCM composites are synthesized by adding 3 wt‰ mBPs in the encapsulation process. Figure 3b,c, illustrate successful encapsulation of the mBPs‐MPCM composites as exemplified by the inner core in the cross‐section of the mBPs‐MPCM composite. The smooth and clean surface suggest that the mBPs are embedded in the core. Figure 3d shows that the size of the mBPs‐MPCM composites ranges from 5 to 30 µm with a mean diameter of about 20 µm. EDS reveals that the mBPs‐MPCM composites are composed of C, O, and P (Figure 3e–h) and the elemental maps disclose uniform distributions of C, O, and P in the mBPs‐MPCM composites. The FTIR spectra of the eicosane, MPCM, and mBPs‐MPCM composites are presented in Figure 3i. Characteristic bands arising from eicosane including rocking vibration of C—H at 720 cm^−1^, vibration of C—C at 892 and 1064 cm^−1^, symmetric bending vibration of methyl CH_3_ at 1376 cm^−1^, group bending vibration of methylene group CH_2_ at 1473 cm^−1^, symmetric stretching vibration of CH_2_ at 2848 cm^−1^, asymmetric stretching vibration of CH_2_ at 2912 cm^−1^, and asymmetric stretching vibration of CH_3_ at 2955 cm^−1^ are observed from the samples. The characteristic peaks of the PMMA shell at 1730, 1243, and 1145 cm^−1^ are associated with the bending vibration of carbonyl and ester group stretching.^[^ ^31^ ^]^ The absence of peaks from mBPs in the FTIR spectra of the mBP‐MPCM samples is probably due to the weak signal from the core. The Raman scattering spectra of the mBPs‐MPCM composites in Figure 3j show the three phonon modes of BP of A^1^_g_, B_2g_, and A^2^_g_ but they cannot be detected from the MPCM composites. The characterization results reveal that mBPs and eicosane are included in the core of the PMMA microencapsulated structure.

**Figure 3:**
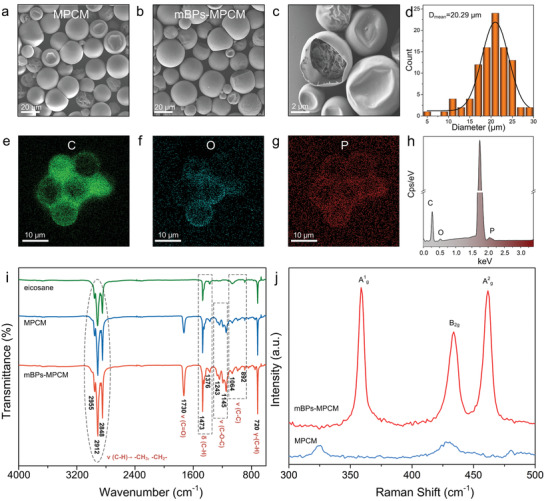
SEM images of the MPCM composites synthesized without and with mBPs: a) MPCM composite and b) mBPs‐MPCM composite; c) Cross‐sectional image of the mBPs‐MPCM composite; d) Statistical analysis of the mBPs‐MPCM composites; e–h) EDS elemental maps and spectrum of the mBPs‐MPCM composites; i) FTIR spectra of the eicosane, MPCM, and mBPs‐MPCM composites; j) Raman scattering spectra of the MPCM and mBPs‐MPCM composites.

The thermal properties of the mBPs‐MPCM composites are investigated with the eicosane and MPCM composites as the control. The differential scanning calorimetry (DSC) spectra are shown in **Figure** **4**a and calculated results are presented in Table S1, Supporting Information. In the dynamic heating and cooling scans, the peaks denoting melting and crystallization can be observed. The melting temperature and crystallization temperature decrease due to the small internal space in the microcapsules.^[^ ^32^ ^]^ Table S1, Supporting Information shows increase/decrease in the melting/freezing temperature and a wide temperature range for the phase transition in the composites compared to pure eicosane perhaps due to the heterogeneous nucleating effect during encapsulation.^[^ ^33^ ^]^ Moreover, both the melting latent heat and crystallization latent heat of the composites decrease after encapsulation on account of the smaller eicosane content in the composites. Nevertheless, the composites still show a high thermal energy storage capacity of over 99.5% and the latent heat is above 180 kJ kg^−1^ compared to pure eicosane (≈235 kJ kg^−1^) indicating a high encapsulation efficiency of more than 78%, however, abovementioned method has no advantage to prepare smaller and larger microcapsules because the latent heat decreases greatly which reduces the energy storage ability (Figure S8, Supporting Information). Samples are also prepared by pressing the composites into thin disks (diameter of 8 mm and thickness of 2 mm) to examine the thermal reliability (inset in Figure 4a). The eicosane and MPCM composites show a white color while the mBPs‐MPCM composites are dark grey at room temperature. By heating the samples to 70 °C for 10 min, the pure eicosane melts completely forming a liquid but the encapsulated samples are unchanged without showing any leakage of eicosane. Hence, the results confirm that the PMMA shells provide sufficient mechanical strength for the microencapsulated composites and prevent leakage of the melted eicosane resulting in good thermal reliability. DSC curves over ten heating/cooling cycles of mBPs‐MPCM composites are recorded (Figure S9, Supporting Information), only a small variation can be observed during cooling process, demonstrating a stable cycle behavior. In addition, the mBPs‐MPCM composites have little color change after 6 month storage at ambient condition, indicating the good stability (Figure S10, Supporting Information).

**Figure 4:**
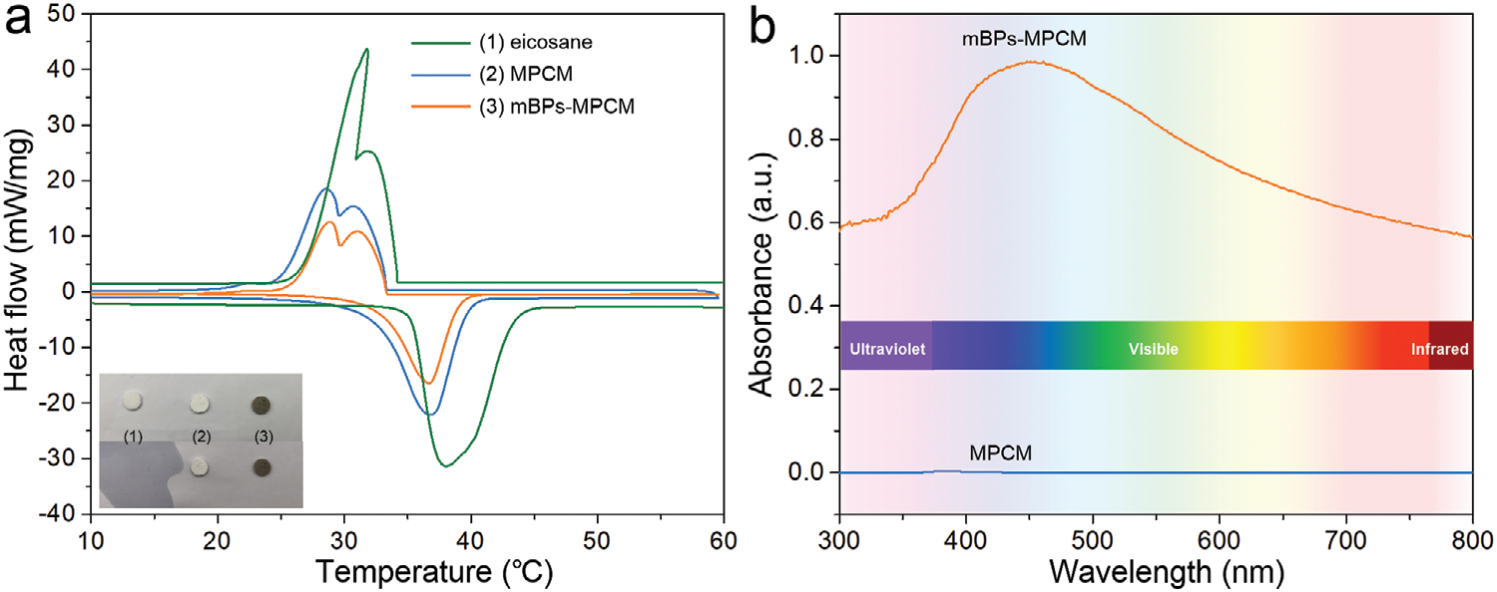
a) DSC curves of (1) eicosane, (2) MPCM composites, and (3) mBPs‐MPCM composites with the inset showing the thermal reliability; b) Absorption spectra of the MPCM and mBPs‐MPCM composites.

The optical absorption properties of the MPCM and mBPs‐MPCM composites are determined by acquiring UV–vis–NIR diffuse reflectance spectra (DRS). As shown in Figure 4b, the absorption spectra from DRS indicate that the MPCM composites without mBPs possess a low capacity for light absorption but on the other hand, the mBPs‐MPCM composites have a much higher capacity for light absorption spanning the UV to NIR regimes, indicating that the favorable optical properties of BPs are preserved in the mBPs‐MPCM composites.

The solar energy storage characteristics of the mBPs‐MPCM composites are investigated. The mBPs decorated MPCM composites with mBPs distributed on the shell surface of the microcapsules are prepared as the control sample (Figure S11, Supporting Information). **Figure** **5**a,b, show the morphology of these two samples confirming the different loading modes of mBPs. Figure 5c displays the photothermal spectra of the mBPs decorated MPCM, mBPs‐MPCM, and pure MPCM samples under illumination by a solar simulator. As the blank controls, the temperature of the pure water and MPCM sample increases slowly indicative of weak light absorption and photothermal conversion (Figure S12, Supporting Information and Figure 5c). In comparison, both the mBPs decorated MPCM and mBPs‐MPCM samples exhibit the photothermal conversion and solar energy storage capabilities. In the mBPs decorated MPCM composites, there are six typical stages in the heating and cooling processes. Upon heating, the temperature of the mBPs decorated MPCM increases quickly from room temperature to 35.3 °C in 510 s followed by a temperature hysteresis stage lasting for 340 s. Afterward, the temperature continues to rise until the solar simulator is turned off. During cooling, the temperature falls to 35 °C and another temperature hysteresis stage lasting for 410 s is observed. Comparing to mBPs decorated MPCM, similar heating and cooling curves are observed from the mBPs‐MPCM composites. However, just a time of 180 s is needed to reach 35.3 °C. The first temperature hysteresis stage lasts for only 100 s and the second temperature hysteresis stage lasts for 600 s. The two temperature plateaus of both samples can be ascribed to the storage of photothermal energy and release of latent heat as a result of the phase change in the inner eicosane. The total energy storage process which includes the heating process to the phase‐changing point and phase‐changing process needs 280 s for mBPs‐MPCM and 850 s for mBPs decorated MPCM, implying that mBPs‐MPCM is three times more efficient pertaining to solar energy storage than mBPs decorated MPCM. The heating rates are calculated to be 0.022 (slop1) and 0.023 (slop2) for mBPs‐MPCM and 0.016 (slop3) and 0.005 (slop4) for mBPs decorated MPCM. The larger heating rates of mBPs‐MPCM may be due to the special thermal conduction. Moreover, with the increased amount of mBPs, the mBPs‐MPCM composites show higher temperature in the same radiation time (Figure S13, Supporting Information).

**Figure 5:**
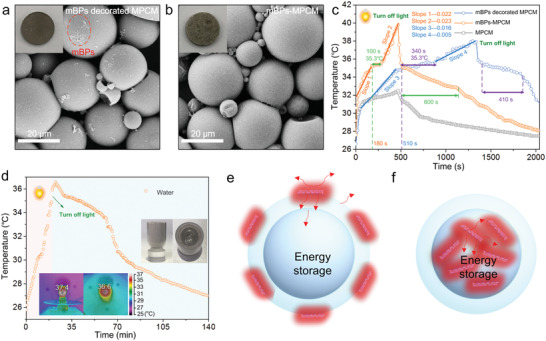
a) SEM image of the mBPs decorated MPCM composites with the inset showing the white light photograph of the pressed tablet; b) SEM image of the mBPs‐MPCM composites with the inset showing the white light photograph of the tablet; c) Photothermal characteristics of the mBPs decorated MPCM, mBPs‐MPCM, and MPCM composites; d) Photothermal characteristics of the cup filled with the mBPs‐MPCM composites with the inset showing the white light photographs and infrared images; e,f) Solar energy storage mechanism of the mBPs decorated MPCM and mBPs‐MPCM composites.

To further investigate the solar energy storage efficiency of the mBPs‐MPCM composites, a cup with a sandwiched structure is designed. The mBPs‐MPCM composites are embedded in the sandwiched layer and the cup is filled with water and irradiated by a solar simulator. The heating and cooling curves as well as infrared images are recorded. As shown in Figure 5d, the mBPs‐MPCM composites are heated to 37.4 °C and water is heated to 36.6 °C in 22 min. Owing to the solar energy storage and release effects, the water temperature is higher than 33 °C for over 40 min under ambient conditions, clearly verifying that the mBPs‐MPCM composites are efficient in solar energy storage.

According to the aforementioned results, the solar energy storage mechanism is postulated. As shown in Figure 5e,f, when the mBPs are outside the MPCM composites, solar energy generates heat by the mBPs and energy transfer/storage occurs in the core/shell structure, surrounding medium of the mBPs, as well as shell. Due to the heat convection between the structure surface and the surrounding medium, a portion of the heat is transferred to the adjacent medium from the mBPs/shell and only a small part of the heat is finally transferred to eicosane. The heat dissipation hampers the energy utilization and storage. The situation changes if mBPs are embedded in eicosane as the heat source without contacting the outside environment and thus, the heat convection between the structure surface and the surrounding medium is minimized. In this case, the generated heat is immediately transferred to eicosane without dissipation as a result of the direct contact between the mBPs and eicosane and therefore, the mBPs‐MPCM composites exhibit enhanced efficiency in harnessing solar energy and swift energy storage, which can also be validated by simulating the simplified equivalent models (Figure S14, Supporting Information) with a result of higher temperature in mBPs‐MPCM than mBPs decorated MPCM.

In summary, the BPs‐incorporated phase‐changing microcapsules deliver efficient solar energy storage performance. Covalent modification of the BPs with PMMA molecules facilitate encapsulation of mBPs and eicosane with PMMA to form a microcapsule structure via the simple emulsion and evaporation method. The mBPs‐MPCM composites exhibit a high latent heat of more than 180 kJ kg^−1^, good thermal reliability, and enhanced solar light absorption after BPs incorporation. During solar irradiation, the mBPs‐MPCM composites show excellent photothermal characteristics including a large heating rate as well as quick phase change resulting in three times higher efficiency in solar energy storage compared to mBPs decorated MPCM. The BPs‐incorporated MPCM composites described here have great potential in solar energy storage applications and the strategy of designing the microcapsules provides insights into the development of multifunctional PCMs.

## Experimental Section

##### Materials

The black phosphorus (BP) powder was purchased from a commercial supplier (Mophos) and stored in a dark Ar glovebox. NMP (≥99.5%), dichloromethane (CH_2_Cl_2_, ≥99.8%), and PVA (MW 67 000) were acquired from Aladdin Ltd. (Shanghai, China) and eicosane (≥99%), and PMMA were obtained from Macklin Reagents (Shanghai, China). All the chemicals were used without further purification.

##### Synthesis of PMMA‐Modified BP Nanosheets (mBPs)

The mBPs were synthesized by a one‐step tip ultrasonic method. In brief, 250 mg of the BP powder were dispersed in 250 mL of the NMP solvent and then mixed with a solution containing of PMMA (0.8 g) and CH_2_Cl_2_ (5 mL). It was sealed at the tip by a Teflon tap and sonicated (ultrasonic frequency, 40 kHz; power, 1200 W) for 8 h (period of 2 s with an interval of 4 s) in an ice bath using a power of 1200 W. The exfoliated solution was centrifuged for 15 min at 4000 rpm and the supernatant was collected following centrifugation for 20 min at 12 000 rpm. The final solution containing the mBPs was collected for further use.

##### Preparation of Microencapsulated Eicosane (MPCM Composites)

0.375 g of PVA were dissolved in 50 g of hot deionized water for 15 min under agitation at 300 rpm. Different amounts of PMMA and eicosane were pre‐dissolved in CH_2_Cl_2_. The experimental groups were designated according to the core/shell ratios (Ratio of 8, 3 g eicosane + 0.375 g PMMA; Ratio of 10.7, 4 g eicosane + 0.375 g PMMA; Ratio of 13.3, 5 g eicosane + 0.375 g PMMA; Ratio of 7.5, 3 g eicosane + 0.4 g PMMA; Ratio of 6, 3 g eicosane + 0.5 g PMMA; Ratio of 4.4, 3 g eicosane + 0.675 g PMMA.). The mixture was added to the PVA solution and stirred vigorously at 3000 rpm for 30 min using a mechanical homogenizer at the ambient temperature to form a stable O/W emulsion. The emulsion was kept at 50  °C under agitation at 300 rpm overnight to evaporate CH_2_Cl_2_. The products were purified by filtration and rinsed thoroughly with warm deionized water and ethanol (≈50 °C). Finally, the MPCM composites were dried at 37 °C for 24 h.

##### Preparation of Microencapsulated Eicosane and mBPs (mBPs‐MPCM Composites)

0.375 g of PVA were dissolved in 50 g of hot deionized water for 15 min under agitation at 300 rpm. At the same time, 3 wt‰ of mBPs was mixed with pre‐dissolved PMMA (0.375g) and eicosane (3 g) in CH_2_Cl_2_ to form a stable oil‐phase solution. The mixture was added to the PVA solution and stirred vigorously at 3000 rpm for 30 min using a mechanical homogenizer at ambient temperature to form a stable O/W emulsion. The emulsion was kept at 50  °C under agitation at 300 rpm overnight to evaporate the CH_2_Cl_2_. The product was purified by filtration and washed thoroughly with warm deionized water and ethanol (≈50 °C). The microcapsules were dried at 37 °C for 24 h.

##### Preparation of mBPs Decorated MPCM Composites

The MPCM composites were prepared with 3 g eicosane and 0.375 g PMMA and 3 wt‰ mBPs was mixed with the MPCM composite powder to form the mBPs decorated MPCM composites.

##### Characterization

SEM was carried out on the ZEISS SUPRA 55 (Carl Zeiss, Germany) field‐emission scanning electron microscope and the EDS data were acquired at an accelerating voltage of 5.0 kV. TEM and high‐resolution transmission electron microscopy (HR‐TEM) were performed on the FEI Tecnai G2 F30 transmission electron microscope at an acceleration voltage of 200 kV. AFM was conducted on the drop‐cast flakes on the SiO_2_ substrate using the Bruker Dimension Icon atomic force microscope (Bruker, USA). Fourier‐transfer infrared (FTIR) spectroscopy was carried out on the PerkinElmer Frontier with the ATR accessory and XPS was performed on the Thermo Fisher ESCALAB 250Xi. The absorption spectra were obtained on the Lambda25 UV–vis–NIR spectrophotometer and DRS data were acquired on the Shimazu UV‐2450 spectrophotometer. Raman scattering was conducted on the Horiba Jobin Yvon LabRam HR‐VIS high‐resolution confocal Raman microscope equipped with a 633 nm laser. The DSC curves were obtained on the TA Instruments Q20 differential scanning calorimeter at a scanning rate of 5 °C min^−1^ from 0 to 60 °C under nitrogen at a flow rate of 50 mL min^−1^. To evaluate the photothermal properties, the temperature change was monitored by an infrared thermal imaging camera (Fluke Ti27, USA).

## Conflict of Interest

The authors declare no conflict of interest.

## Supporting information

Supporting Information
