# Supplementary material for: Phase‐Changing Microcapsules Incorporated with Black Phosphorus for Efficient Solar Energy Storage
Source: Adv Sci (Weinh). 2020 Oct 22;7(23):2000602. doi: 10.1002/advs.202000602 (PMC7709984; doi:10.1002/advs.202000602)
Supplement: Supplementary file 1 — Supporting Information [file ADVS-7-2000602-s001.pdf]

© 2020 Wiley-VCH GmbH

ADVANCED SCIENCE
Open Access

Supporting Information

for *Adv. Sci.*, DOI: 10.1002/advs.202000602

Phase-Changing Microcapsules Incorporated with Black Phosphorus for Efficient Solar Energy Storage

*Hao Huang, Tongyu Shi, Rui He, Jiahong Wang, Paul K. Chu, and Xue-Feng Yu**

Supporting Information

Phase-Changing Microcapsules Incorporated with Black Phosphorus for Efficient Solar Energy Storage

*Hao Huang, Tongyu Shi, Rui He, Jiahong Wang, Paul K. Chu, and Xue-Feng Yu**

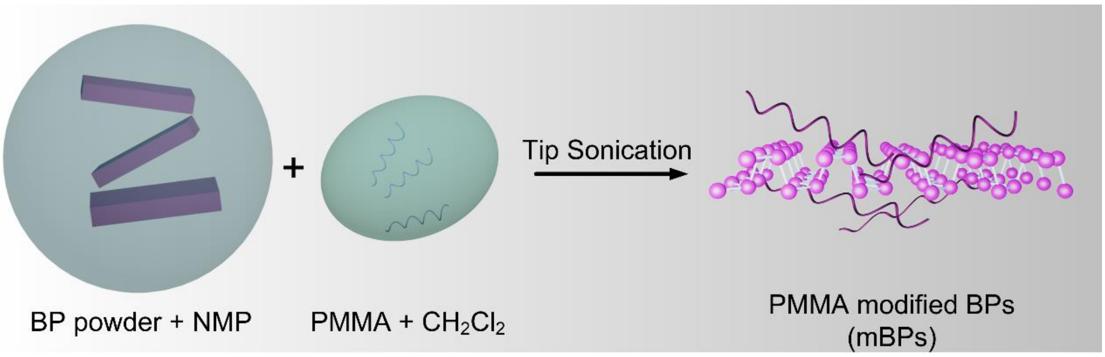

Figure S1. Synthesis of mBPs.

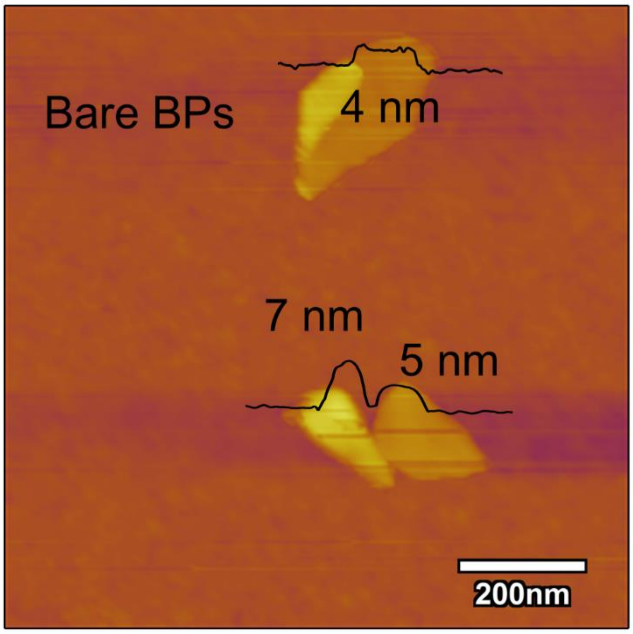

Figure S2. AFM image of bare BPs.

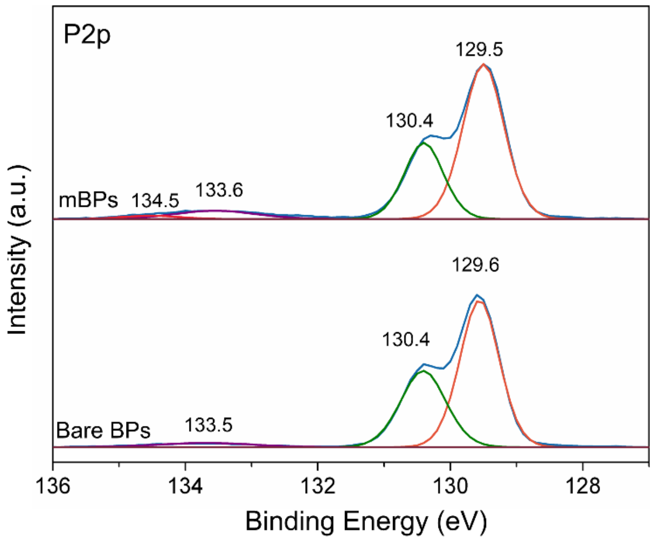

Figure S3. XPS P 2p spectra of bare BPs and mBPs.

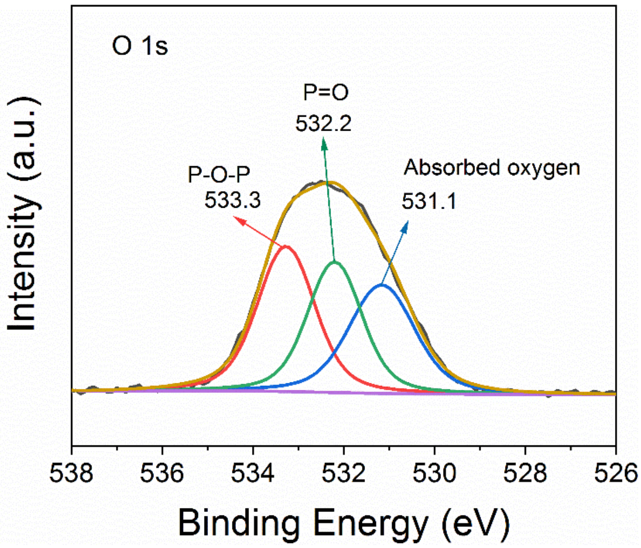

Figure S4. XPS O 1s spectra of bare BPs.

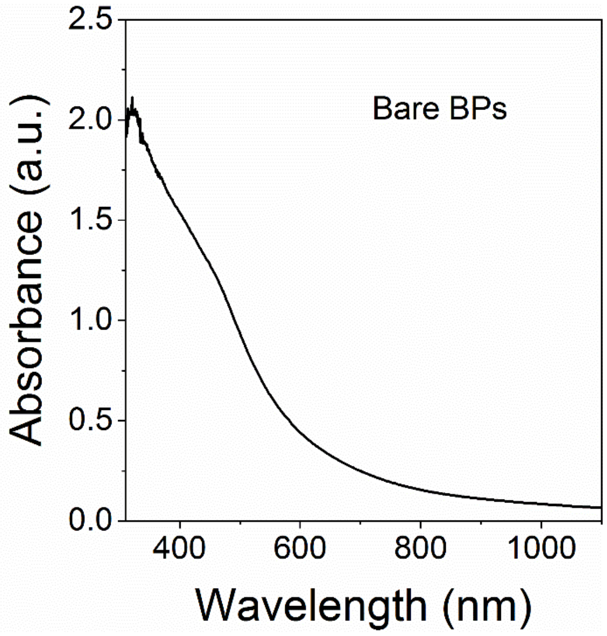

Figure S5. Absorption spectrum of bare BPs in NMP.

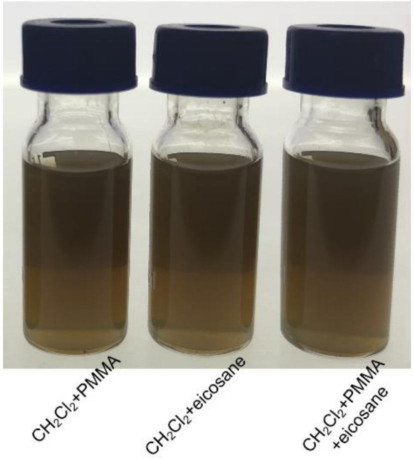

Figure S6. The dispersibility of mBPs in CH_2Cl_2 + PMMA, CH_2Cl_2 + eicosane and CH_2Cl_2 + PMMA + eicosane.

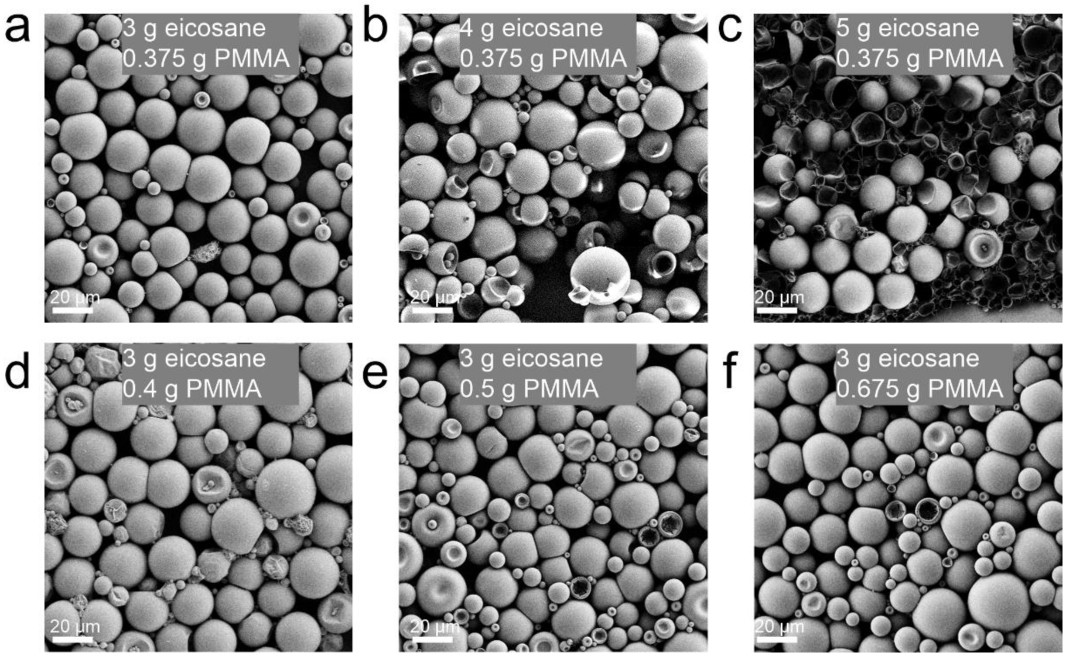

Figure S7. SEM images of the MPCM composites with different core/shell ratios. (a) Ratio of 8, 3 g eicosane + 0.375 g PMMA; (b) Ratio of 10.7, 4 g eicosane + 0.375 g PMMA; (c) Ratio of 13.3, 5 g eicosane + 0.375 g PMMA; (d) Ratio of 7.5, 3 g eicosane + 0.4 g PMMA; (e) Ratio of 6, 3 g eicosane + 0.5 g PMMA; (f) Ratio of 4.4, 3 g eicosane + 0.675 g PMMA.

Table1. Phase-changing characteristics and encapsulation parameters of the microcapsule samples.

Sample	Melting temperature (°C)	Melting latent heat (kJ/kg) ΔH_m	Cooling latent heat (kJ/Kg) ΔH_C	Encapsulation ratio (%) R	Encapsulation efficiency (%) E	Thermal storage Capability (%) η
eicosane	38.00	235.30	235.40			
MPCM	36.82	200.70	200.20	85.29	85.17	99.85
mBPs-MPCM	36.71	186.60	185.10	79.30	78.97	99.58

The encapsulation parameters are calculated by following equations:

$$\mathrm{R}(\%) = \frac{\Delta H_{m,Sphere}}{\Delta H_{m,Eicosane}} \times 100$$

$$\mathrm{E}(\%) = \frac{\Delta H_{m,Sphere} + \Delta H_{C,Sphere}}{\Delta H_{m,Eicosane} + \Delta H_{C,Eicosane}} \times 100$$

$$\eta(\%) = \frac{\Delta H_{m,Eicosane}(\Delta H_{m,Sphere} + \Delta H_{C,Sphere})}{\Delta H_{m,Sphere}(\Delta H_{m,Eicosane} + \Delta H_{C,Eicosane})} \times 100$$

Where $\Delta H_{m,Eicosane}$ and $\Delta H_{m,Sphere}$ are the melting latent heat of the eicosane and two types of microcapsules and $\Delta H_{C,Eicosane}$ and $\Delta H_{C,Sphere}$ represent the cooling latent heat of the eicosane and microcapsules, respectively.

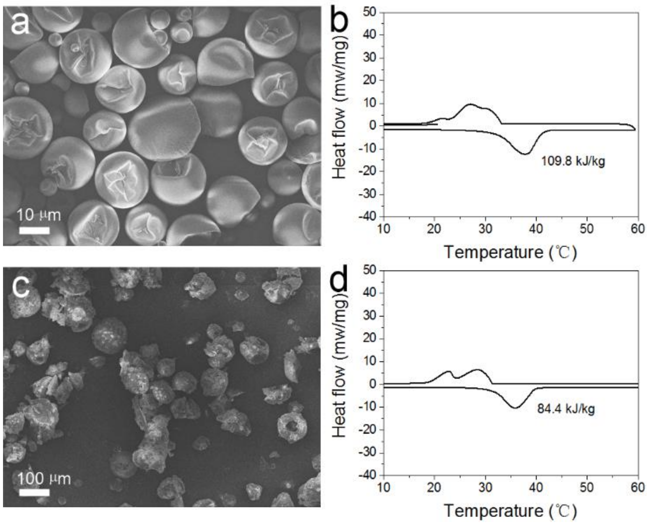

Figure S8. SEM images and DSC curves of smaller (a, b) and larger (c, d) microcapsules.

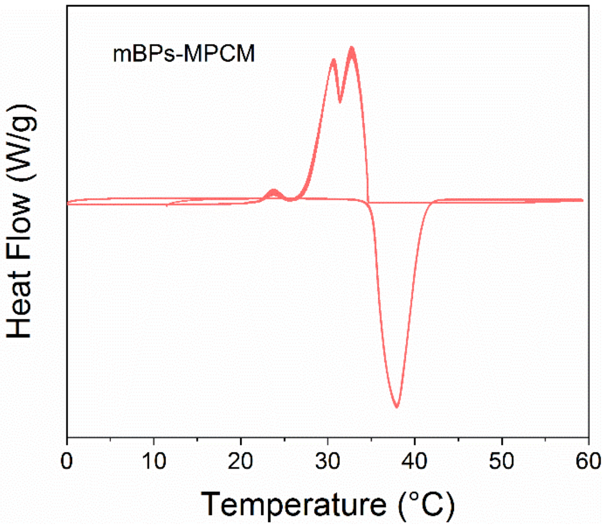

Figure S9. DSC thermogram over ten heating/cooling cycles of mBPs-MPCM composites.

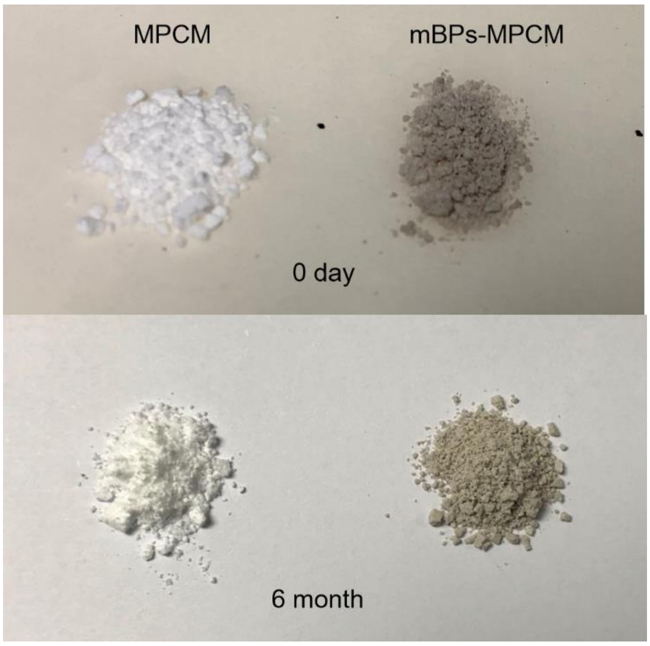

Figure S10. The stability of mBPs-MPCM composites at ambient condition.

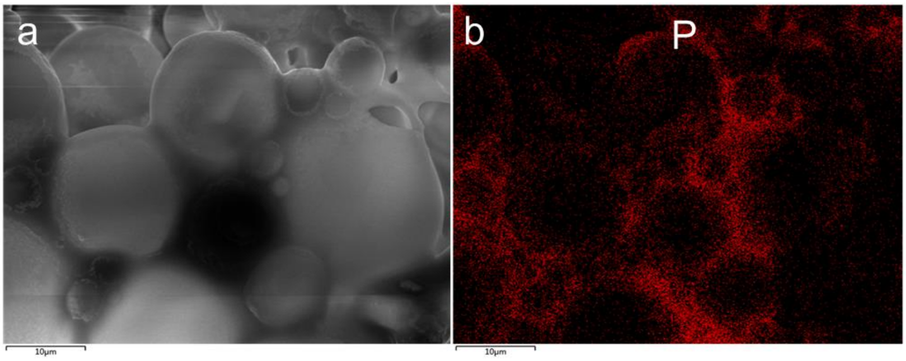

Figure S11. EDS maps of mBPs decorated MPCM composites.

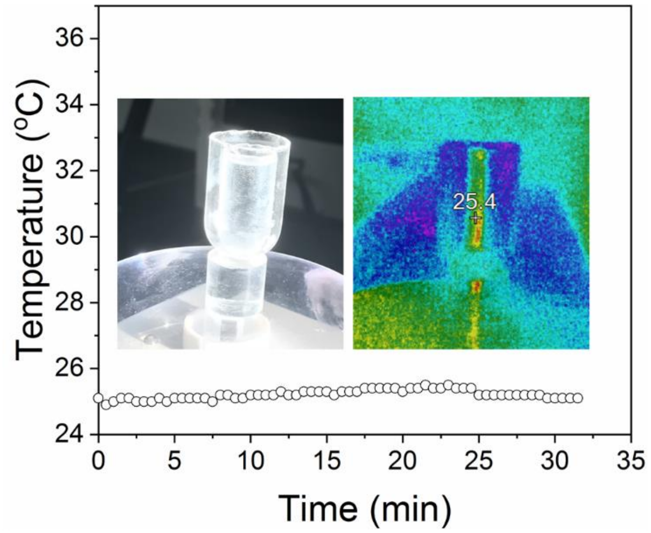

Figure S12. Photothermal characteristics of pure water.

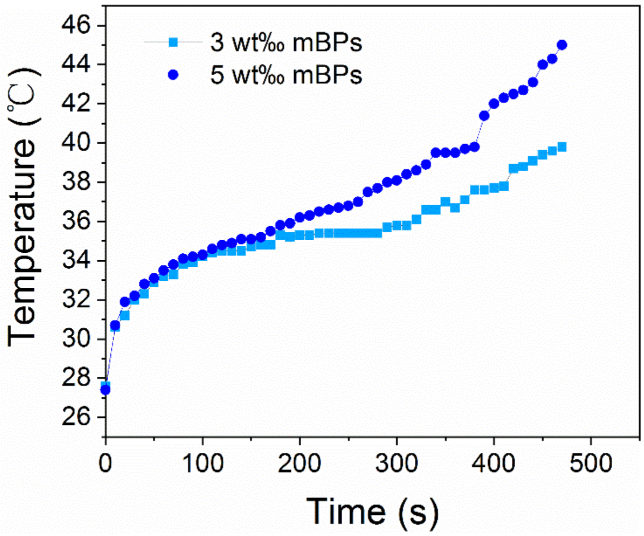

Figure S13. Photothermal performance of mBPs-MPCM composites prepared with 3wt‰ and 5wt‰ of mBPs.

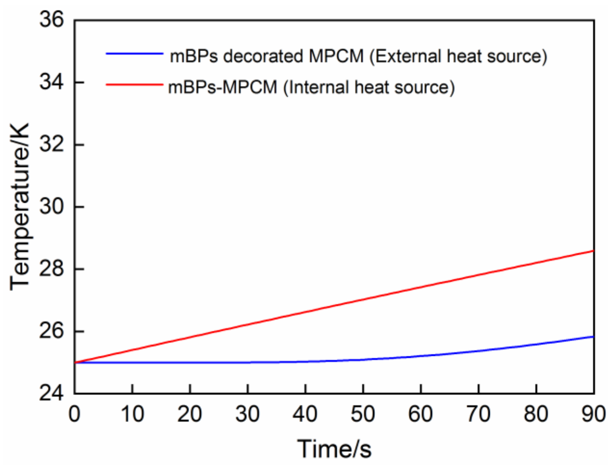

Figure S14. Model simulation. Variation of temperature of core material with time.

Model Description

According to our experiments, the simulation of the mBPs decorated MPCM and mBPs-MPCM composites for solar-energy storage is simplified as sphere one-dimensional heat conduction. The models for mBPs decorated MPCM and mBPs-MPCM composites are shown below. In the models the mBPs are served as heat sources and thus, external and internal heat sources are applied to mBPs decorated MPCM and mBPs-MPCM models, respectively.

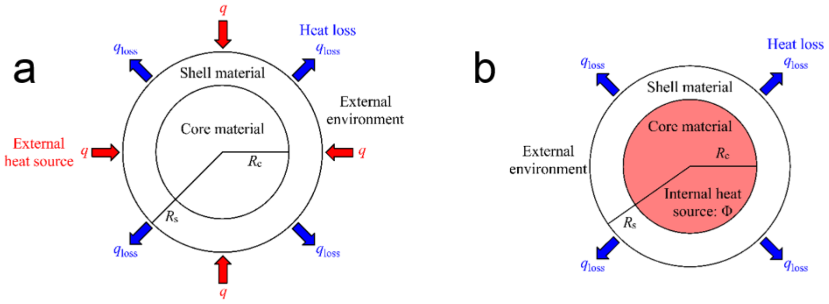

Fig. 1 Physical model of the heat conductivity process of a single microcapsule. (a) Model for mBPs decorated MPCM (External heat source); (b) Model for mBPs-MPCM (Internal heat source). Where the q (W·m^{-2}), q_{loss} (W·m^{-2}), Φ (W·m^{-3}) and E (W·m^{-2}) represent heat flux of external heat source, heat loss to external environment, source term of internal heat source and the power density of solar radiation, respectively.

Hypothesis:

1) The heat conduction in the sphere is rotation symmetric of the spherical center.
2) The heat source and heat loss are rotation symmetric of the spherical center.
3) The heat energy generated by internal heat source is uniformly distributed in the core.
4) The physical parameters of the core and shell are not changed with temperature.

With the light radiation, q and Φ can be calculated according equations (1) and (2):

$$q = \frac{E \cdot \pi R_s^2}{4\pi R_s^2} = \frac{E}{4} \qquad (1)$$

$$\Phi = \frac{E \cdot \pi R_c^2}{\frac{4}{3}\pi R_c^3} = \frac{3E}{4R_c} \qquad (2)$$

According to symmetry hypothesis, the heat conduction in Fig.1 can be simplified as one-dimensional heat conduction in spherical coordinates. The heat conduction equation (3) is shown below [1].

$$\rho c \frac{\partial T}{\partial t} = \frac{1}{r^2} \frac{\partial \left(\lambda r^2 \frac{\partial T}{\partial r} \right)}{\partial r} + S \qquad (3)$$

Where the ρ (kg·m^{-3}), T (℃), t (s), r (m), c (J·kg^{-1}·K^{-1}), λ (W·m^{-1}·K^{-1}) and S (W·m^{-3}) are the density, the temperature, the time, the radial coordinate, the specific heat, the thermal conductivity and the source term, respectively. S=Φ (Internal heat source), S=0 (External heat source).

Numerical Model

Uniform cell centered scheme is used.

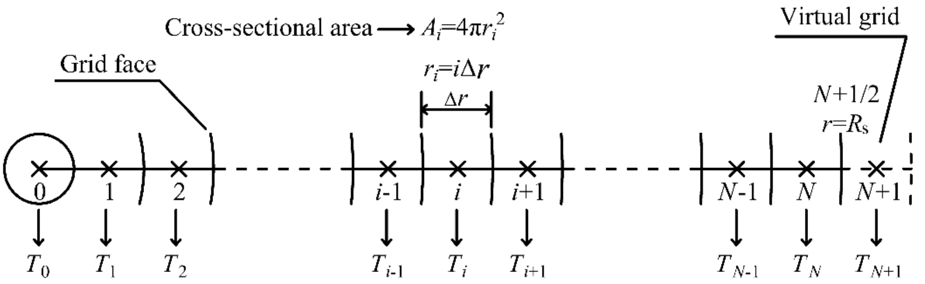

Fig.2 Sketch of the grid partition.

Where the i, Δr, r_i, T_i, 1/2 and N+1 are the grid number, the grid step at i, the temperature at i, the grid interface and the virtual grid, respectively.

Discrete Equations

Based on conservation of energy, the discrete equation of heat conduction can be derived. The heat of grid i introduced from the interface of i-1/2 and i+1/2 can be calculated according to following equations:

$$q_{i-1/2} = \lambda \cdot 4\pi r_{i-1/2}^2 \frac{T_{i-1} - T_i}{\Delta r} \qquad (4)$$

$$q_{i+1/2} = \lambda \cdot 4\pi r_{i+1/2}^2 \cdot \frac{T_{i+1} - T_i}{\Delta r} \qquad (5)$$

In which r_i can be calculated:

$$r_i = i \cdot \Delta r \qquad (6)$$

Heat from internal heat source:

$$q_s = S_i \cdot V_i \qquad (7)$$

The grid volume V_i:

$$V_i = 4\pi r_i^2 \cdot \Delta r \qquad (8)$$

Heat variation of grid i in unit time, where the Δt, n and n+1 are the time step, the current and next time step number:

$$q_t = \rho V_i c \frac{T_i^{n+1} - T_i^n}{\Delta t} \qquad (9)$$

Based on conservation of energy,

$$q_t = q_{i-1/2} + q_{i+1/2} + q_s \qquad (10)$$

According to equations (4) to (10), the discrete equation of heat conduction at grid i can be derived:

$$T_i^{n+1} - T_i^n = \frac{\lambda}{\rho c}\frac{\Delta t}{\Delta r^2}\left(\left(1 - \frac{1}{2i}\right)^2\left(T_{i-1}^{n+1} - T_i^{n+1}\right) + \left(1 + \frac{1}{2i}\right)^2\left(T_{i+1}^{n+1} - T_i^{n+1}\right)\right) + \frac{\Delta t}{\rho c}S_i \qquad (11)$$

Further simplified:

$$A_i T_{i-1}^{n+1} + B_i T_i^{n+1} + C_i T_{i+1}^{n+1} = T_i^n + T_i^* \qquad (12)$$

Where the A_i、B_i、C_i and T^* can be calculated:

$$A_i = -\frac{\lambda}{\rho c}\frac{\Delta t}{\Delta r^2}\left(1 - \frac{1}{2i}\right)^2 \qquad (13)$$

$$C_i = -\frac{\lambda}{\rho c}\frac{\Delta t}{\Delta r^2}\left(1 + \frac{1}{2i}\right)^2 \qquad (14)$$

$$B_i = 1 - \left(A_i + C_i\right) \qquad (15)$$

$$T_i^* = \frac{\Delta t}{\rho c}S_i \qquad (16)$$

Boundary Conditions

In the calculation in Fig.2, two boundaries r=0 and r=R_s exist, and R_s can be calculated by equation:

$$R_s = \left(N + \frac{1}{2}\right)\Delta r \qquad (17)$$

The grid volume V_0 at boundary r=0:

$$V_0 = \frac{4\pi}{3}\left(\frac{1}{2}\Delta r\right)^3 \tag{18}$$

Considering there is only right boundary at boundary r=0 and equation (4),

$$\rho c \cdot \frac{4\pi}{3}\left(\frac{1}{2}\Delta r\right)^3 \cdot \frac{T_0^{n+1} - T_0^{n}}{\Delta t} = \lambda \cdot 4\pi\left(\frac{1}{2}\Delta r\right)^2 \cdot \frac{T_1^{n+1} - T_0^{n+1}}{\Delta r} + \Phi_i \cdot \frac{4\pi}{3}\left(\frac{1}{2}\Delta r\right)_i^3 \tag{19}$$

Further simplified:

$$B_0 T_0^{n+1} + C_0 T_1^{n+1} = T_0^{n} + T_0^{*} \tag{20}$$

In addition,

$$C_0 = -\frac{6\lambda}{\rho c}\frac{\Delta t}{\Delta r^2} \tag{21}$$

$$B_0 = 1 - C_0 \tag{22}$$

The relationship between the virtual node temperature (T_{N+1}) and internal node temperature at External boundary r=R_s should be considered. There is heat transfer between the shell and external environment, including the heat from external heat source q and heat loss q_{loss}.

$$q_{loss} = h_f\left(T_f - T_{N+1/2}\right) \tag{23}$$

Where the h_f and T_f are the convective heat transfer coefficient and temperature of the environment.

Relationship at boundary N+1/2:

$$q_{loss} + q = \lambda\frac{T_{N+1} - T_N}{\Delta r} \tag{24}$$

$$T_{N+1/2} = \frac{T_N + T_{N+1}}{2} \tag{25}$$

Based on equations (23) to (25),

$$T_{N+1}^{n+1} = \frac{1 - \frac{1}{2}\frac{h_f \Delta r}{\lambda}}{1 + \frac{1}{2}\frac{h_f \Delta r}{\lambda}} T_N^{n+1} + \frac{\frac{h_f \Delta r}{\lambda} T_f + \frac{q \Delta r}{\lambda}}{1 + \frac{1}{2}\frac{h_f \Delta r}{\lambda}} \tag{26}$$

The convective heat transfer coefficient can be calculated, where the D_s is the diameter of the microcapsule.

$$h = Nu\frac{\lambda}{D_s} \tag{27}$$

The Nu is the Nussel number of natural convective heat transfer. The Ra and Pr are Rayleign number and Prandtl number of the air.

$$Nu = 2 + \frac{0.589 Ra^{1/4}}{\left(1 + \left(\frac{0.469}{Pr}\right)^{9/16}\right)^{4/9}} \quad (28)$$

The physical parameters involved in the calculation are shown in Table 1. And the output temperatures are acquired from the outside and center of the core for internal heat source (mBPs-MPCM) and external heat source (mBPs decorated MPCM) group, respectively.

Table 1 Parameters for calculation

Parameters	Value	
	Core material	Shell material
ρ (kg·m^{-3})	900	1180
λ (W·m^{-1}·K^{-1})	0.12	0.18
c (J·kg^{-1}·K^{-1})	3200	1400
Radius (mm)	5.94	6.0
E (W·m^{-2})	1000	
Ambient temperature/℃	25.0	
Ambient pressure/kPa	101.325	
Initial temperature/℃	25.0	

1. Theodore L. Bergman, Adrienne S. Lavine, Frank P. Incropera, David P. Dewitt. Fundamentals of heat and mass transfer [M]. John Wiley & Sons, 7th edition, 2011, 83-87, 594-617.
